# Games and Health Education for Diabetes Control: A Systematic Review with Meta-Analysis

**DOI:** 10.3390/healthcare8040399

**Published:** 2020-10-14

**Authors:** María Begoña Martos-Cabrera, María José Membrive-Jiménez, Nora Suleiman-Martos, Emilio Mota-Romero, Guillermo Arturo Cañadas-De la Fuente, José L. Gómez-Urquiza, Luis Albendín-García

**Affiliations:** 1 San Cecilio Clinical University Hospital, Andalusian Health Service, Avenida del Conocimiento s/n, 18016 Granada, Spain; mbmartos@ujaen.es; 2 Ceuta University Hospital, National Institute of Health Management, Loma del Colmenar s/n, 51003 Ceuta, Spain; mariajosemembrive@correo.ugr.es; 3 Faculty of Health Sciences, Ceuta University Campus, University of Granada, C/Cortadura del Valle SN, 51001 Ceuta, Spain; norasm@ugr.es; 4 Assitance Unit Doctores Dr. Salvador Caballero García, Granada-Metropolitano Health District, Andalusian Health Service, C/Joaquina Eguaras, n° 2, Edificio 2, 1ᵃ planta, 18013 Granada, Spain; emilio.mota.sspa@juntadeandalucia.es; 5 Faculty of Health Sciences, University of Granada, Avenida de la Ilustración N. 60, 18016 Granada, Spain; gacf@ugr.es; 6 Assitance Unit La Chana, Granada-Metropolitano Health District, Andalusian Health Service, C/Joaquina Eguaras, n° 2, Edificio 2, 1ᵃ planta, 18013 Granada, Spain; lualbgar1979@ugr.es

**Keywords:** diabetes mellitus, disease management, games, gamification, glycated hemoglobin A, meta-analysis, smartphone, systematic review

## Abstract

Finding methods to improve people’s diabetes control and management is important to prevent its complications and maintain the quality of life. The aim of this review was to assess the effect of games on the blood glucose level (glycated hemoglobin (HbA1c)). A systematic review and meta-analysis were made. Pubmed, Scopus, and CINAHL databases were consulted in July of 2020. Ten studies were selected as a final sample, most of them being clinical trials using games to improve diabetes control. Half of the studies had samples between 8 and 14.9 years old and the other half between 57 and 65 years old. The studies informed about using applications/games for mobile phones, game consoles, and board games for diabetes education and management. The meta-analysis was performed with 4 studies showing a mean difference of 0.12 (CI 95% 0.57, 0.33) of HbA1c in favor of the intervention group with *p* > 0.05. Games are positive for diabetes health education and promoting healthier lifestyle, but their impact on HbA1c is low.

## 1. Introduction

Diabetes Mellitus (DM) is an endocrine disease, with a certain hereditary component, characterized by an increase in blood glucose. It can be classified into type 1 (DM1) or insulin-dependent diabetes, type 2 (DM2) or non-insulin-dependent diabetes mellitus, and others less frequent such as gestational diabetes and sub-categories of DM1 like Latent Autoimmune Diabetes in Adults (LADA) and Maturity Onset Diabetes of Youth (MODY) [1,2].

DM1 is characterized by autoimmune B-cell destruction, which generally leads to absolute insulin deficiency, and it usually occurs in children or young adults. The characteristic symptoms are: polyuria, polydipsia, and, approximately a third, present diabetic ketoacidosis. In DM1, there are no factors that can be prevented, but fluctuations in blood glucose must also be controlled to avoid hyperglycemia and hypoglycemia. A good control of blood glucose will improve the quality of life and will avoid secondary pathologies [3].

On the other hand, DM2 is a chronic disease characterized by an increase in blood glucose. This is due to a relative insulin deficiency caused by pancreatic β-cell dysfunction and insulin resistance in target organs [3,4]. In DM2, there are risk factors that influence its development and evolution such as overweight, sugar consumption, or sedentary life [5]. For example, being overweight or obese can cause some degree of insulin resistance, which is present in many people with diabetes in their adulthood [3,6].

Glucose level control is essential to decrease the risk of diabetes microvascular and macrovascular complications [3]. According to recent data, 75% of DM2 cases develop in low and middle-income countries, and total global health spending due to diabetes is estimated at 673 billion dollars [3,4,7]. Furthermore, according to the “World Diabetes Report”, this situation is increasing, with approximately 415 millions of people with diabetes in 2015 and an estimated 642 million people by 2040 [4,7].

DM2 and its complications can be prevented by performing physical activity regularly, following a healthy diet, and avoiding tobacco and alcohol, and controlling parameters such as blood pressure and cholesterol [4,8]. Thus, a non-pharmacological approach with healthy habits is essential to improve blood glucose levels, which are reflected in the glycated hemoglobin (HbA1c) values [3,9]. HbA1c is a very reliable measure that averages blood glucose over the past three months [10,11]. Finally, in those cases for which it is necessary, the inclusion of pharmacological treatment with oral antidiabetics and subcutaneous insulin will be chosen [12].

Glucose level control is managed by each person who needs health education and tools like glucometers, smartphone applications, or an insulin pump. Taking this into account, and all the variables that play an important role in diabetes control, it is important to treat people with diabetes from a multidisciplinary view (to improve all the necessary aspects for the control of the disease such as nutrition, treatments, health education, motivation, etc.) and using technology to help. People with diabetes, with the help of new technologies, can significantly improve their HbA1c levels [13,14]. Games and gamification (application of game elements and its dynamics in non-game environments) can be a good instrument for health education and management of chronic diseases, since it is motivating and fun and consequently, more efficient for the learning process and the management of the disease [15,16,17]. The use of games, although their engagement seems to be short-term, is a strong promise for diabetes education and management, but it is necessary to perform more research to clarify its real impact [17,18].

Thus, taking into account the positive influence of games and gamification on behavioral changes related to health and healthy lifestyles [17,19], and the positive influence of these on diseases control and management, the aim of this study was to analyze the effect of games and gamification on the levels of glycated hemoglobin (HbA1c) in people with diabetes.

## 2. Materials and Methods

A systematic review with meta-analysis, following the guidelines of the PRISMA statement [20], was performed.

### 2.1. Elegibility Criteria

Primary studies that analyzed the influence of games and gamification on the levels of HbA1c, with people with DM1 and DM2, published in English and Spanish, without restriction by year of publication and without restriction in the age of the participants were included. Duplicated studies or those with a pre-diabetic population were excluded.

### 2.2. Information Sources and Search

The following databases were consulted: Pubmed, Scopus, and CINAHL. The search equations are indicated in Table 1. The search was completed on 31 July 2020.

### 2.3. Study Selection and Risk of Bias

The results obtained in the databases were first selected by reading the title and abstract. Then, the full text was read. After that, a critical reading was made and finally, a reverse search, reading the references of each study, was performed with the included studies. The selection process was completed independently by two members of the team, consulting a third person in case of disagreement. The critical reading was done with CASPE critical appraisal checklists.

In order to assess the level of evidence and grade of recommendation of the included studies, the Oxford Center for Evidence-based Medicine (OCEBM) [21] recommendations were followed.

### 2.4. Data Items and Collection Process

A data collection notebook was used. The following variables about the characteristics of the sample were collected: year of publication, country of study, study design, sample, game or gamification characteristics, and main results in relation to glycated hemoglobin (HbA1c) and diabetes management.

### 2.5. Data Analysis and Summary of Results

All the studies included in the review were descriptively analyzed and categorized in 3 sections (Electronic and board games for diabetes knowledge acquisition, Electronic simulation games for solving problems and situations related to diabetes, and Electronic games for adherence to physical exercise in people with diabetes). The effect size of the intervention for the reduction of HbA1c (mean difference between control and intervention group with a confidence interval of 95%) was calculated with a random effect meta-analysis done with Review Manager 5.3. For the meta-analysis calculation, the necessary data from each study were the mean, standard deviation, and sample size of control and intervention group. Before the meta-analysis, a sensitivity analysis (to check that the effect size did not significantly change after eliminating each study from the meta-analysis) and an Egger test for publication bias were performed. *I*^2^ test was used for heterogeneity analysis. The Cochrane handbook for systematic reviews of interventions was followed for the analyses [22].

## 3. Results

### 3.1. Study Selection and Study Characteristics

**Figure 1:**
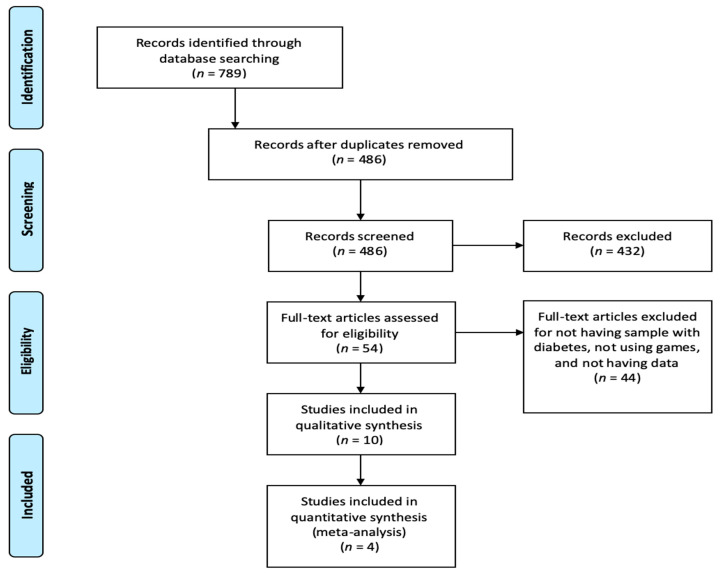
Prisma flow diagram of study selections.

The search showed 789 results that were reduced to 54 after eliminating duplicates and reading the title and abstract. Then, after reading the full texts, 44 studies were eliminated due to the fact that they did not include information about HbA1c, had no people with diabetes sample or were not using games or gamification process, leaving a final sample of *n* = 10 studies [23,24,25,26,27,28,29,30,31,32] (Figure 1).

The 70% of the studies were randomized clinical trials, 10% cohort studies, 10% cases and controls studies, and 10% qualitative studies. Most of the studies were performed in the USA (70%) and others studies were performed in Canada, Switzerland, and France. The higher sample was *n* = 456 and the lower *n* = 20. The 40% of the studies were centered in people with DM1. The main characteristics of the included studies and the results about using games and gamification for diabetes are summarized in Table 2.

### 3.2. Electronic and Board Games for Diabetes Knowledge Acquisition

The use of mobile applications is very widespread, since the smartphone is an essential element in our day to day life. One study with children used an application that automatically received blood glucose levels through Bluetooth [23]. The app generated graphs of blood glucose level evolution and gamified the glucose control giving rewards when reaching healthy goals (keeping blood glucose level in range), such as free downloads of iTunes songs [23]. In a 12-week period, HbA1c did not change significantly, but improved the glycemic control of the sample and 88% of participants assured that they would use the app after the study since they found it useful for making decisions about their health [23].

Another study used the DiaSocial app for 14 weeks to promote adherence to physical exercise, improved nutrition, and adherence to drug therapy in veterans (mean age 65.4 years) with DM2. Training about the app was done in groups and the app gave points depending on people performance of healthy activities (maintaining blood glucose level, recording blood glucose level, adequate nutritional intake, minutes of exercise, and adherence to medication) [24]. Higher final scores were observed in people with greater reduction of HbA1c [24]. The mean HbA1c in the control group, which received a classic health education of recommendations and control, was 8.78 and in the intervention group, it was 8.06 [24].

Other authors tested two games as a method for acquiring knowledge about diabetes in a sample with 59 years old and DM2 [25]. The intervention group received a game about diabetes self-management and a brochure on civic standards. The control group received a game of civic standards and a brochure on diabetes self-management standards. In addition, two questions about diabetes self-management education were sent two days per week through a mobile application or email, and the correct answer and the explanation are shown automatically after the user’s answer. Correct answer gave points to the person and, with the points, a ranking of the participants was elaborated. The intervention group, at 6 months, showed an improvement in HbA1c, but not statistically significant, while empowerment over the disease increased [25].

In the study by Kumar et al., 2004 [26], performed with children and adolescents, a motivational game was used in the intervention group and the participants had the Dia-BetNet software on their personal digital assistant. The software integrates outcomes for diabetes management (previous blood glucose levels, insulin doses, and carbohydrates consumed) and challenges participants to predict their next glucose level based on a graphical display [26]. The participants received points for playing the prediction and more points for its accuracy. The intervention increased the number of blood glucose checks per day, but did not provide a direct improvement in HbA1c, but a decrease in hyperglycemia [26].

Another study used board games to encourage communication between people with diabetes and, with it, the acquisition of knowledge [27]. The study observed how, after playing the board game, participants improved their HbA1c [27]. The intervention was based on different sessions where a “conversation” on a Conversation Map was “played”. People took advantage of the experiences of others participants and also another maps added 4 different educational tools to the previous one: general description of diabetes, healthy eating, monitoring and use of their results, and natural course of diabetes. The intervention lasted 3 years and it demonstrated an improvement in HbA1c in the people who played compared to the people who received classical care. Game players decreased their HbA1c to 6.96 while the control group decreased to 8.27 [27].

### 3.3. Electronic Simulation Games for Solving Problems and Situations Related to Diabetes

Brown et al. [28] used a Super Nintendo Entertainment System^®^ video game. The game was called “Packy and Marlon” and was designed for young people, between 7 and 15 years, with DM1. The game simulated a diabetes camp where the protagonists had to maintain their health through specific behaviors (food choices, doing too little or too much exercise, and what to do according to the circumstances) [28]. This intervention did not significantly improve HbAc1, but knowledge and problem solving improved and it also decreased emergency room visits by 77% [28]. Another game simulation for problem solving in children and adolescents was “L’Affaire Birman” [29]. The protagonist of the game is a teenage boy named Alex who has flexible insulin therapy and the player, through his decisions, must maintain Alex’s health. The intervention did not show a significant improvement in HbA1c, but it did improve knowledge about carbohydrate counting and insulin administration [29].

Another study used the simulation game “Patient Partner” that is designed for maintaining health through real life situations. Contrary to the previous two games, this was a mobile app focused on DM2. The use of this application decreased HbA1c and increased adherence to diet, exercise, and medication [30].

### 3.4. Electronic Games for Adherence to Physical Exercise in People with Diabetes

One study that used a smartphone game showed improvements in 24 weeks in the number of steps per day, but it did not influence a significant modification of HbAc1 [31]. Finally, a study that used a Wii Fit Plus sports game for 12 weeks 30 min a day improved adherence to physical activity and decreased HbAc1d from 7.1% to 6.8% [32].

### 3.5. Meta-Analysis Results

**Figure 2:**
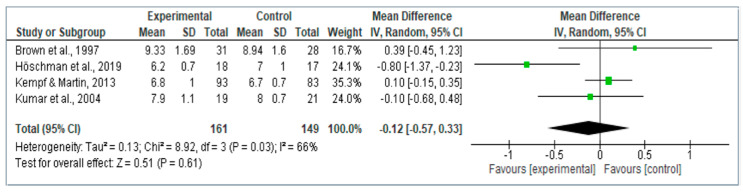
Forestplot of the effect size of games and gamification for glycated hemoglobin (HbA1c) reduction in people with diabetes.

From the ten studies included in the review, only four had the necessary information (mean and standard deviation after the intervention for the control and the intervention group) for the meta-analysis; 50% of the studies had samples with DM2. The sample of the intervention group was *n* = 161 and for the control group, it was *n* = 149. The means differences in the percentage of HbA1c was −0.12 (95% confidence interval −0.57, 0.33) in favor of the intervention group, but without being statistically significant. The forestplot is shown in Figure 2.

## 4. Discussion

The use of game and gamification processes reduces HbA1c level, but it does not show significant differences with the control group. Mobile applications linked to games and gamification processes with educational health content are an advance for people with diabetes, but their use does not make an effective difference with other usual interventions. In the case of DM1, when debuting at an early age, an effective method of communication and transmission of knowledge is by playing [33]. This can help to learn the administration of subcutaneous insulin to children [34] and other things like proper nutrition [35]. Although it seems that the games are more oriented to children and adolescents, there are also games for adults that are effective [36].

Even though various studies demonstrate the effectiveness of using games through mobile applications for health education in people with diabetes [37,38], some authors said that technology can be a barrier for some populations [39]. Research in this field should continue to advance in order to make the use of mobile applications easier and more practical [40], since, in some cases, patients may not learn the basic concepts needed for good self-control of the disease [18,41]. Moreover, health professional training is required to recommend and apply these technologies, knowing that they are safe and reliable [42]. The use of innovative therapies such as the use of games in people with diabetes is a very useful tool in clinical practice [43] for problems’ solutions, since it improves adherence and minimizes complications [44].

In addition, the use of games in mobile applications has recently been implemented. These facilitate access to people with diabetes and are an educational tool that can improve HbA1c levels [45]. Health education provides many benefits to patients [46]. Nonetheless, it is true that it requires a responsibility on the part of the patients [47] and that a standardization and simplification of the different applications is necessary, since there is a decrease in communication with health professionals [48].

Making a person adhere to the recommendations and treatments that professionals give is not easy. It has been observed that patients follow medical prescriptions better if they understand the treatment. Several authors affirm that the integration of technology-based exercise programs can have a positive effect on the adherence of the diabetic patient, since they produce an increase in enjoyment and make it easier to perform these programs [49,50]. Patients think that everything is important and these applications help them make decisions [51]. The use of these methods encourages the user to carry out physical activity, producing favorable results in the medium–long term and leading to an improvement in balance or flexibility [52] as well as an improvement at a psychological level, reducing the levels of depression [53,54]. However, there are authors who state that practicing physical exercise through the use of electronic applications or games only produces psychological benefits and does not improve adherence [55]. Likewise, other authors say that they do not observe this type of benefits, and affirm that adherence is greater when performed outdoors [56]. In turn, some authors claim that these methods are beneficial for exercising continuously from home. Decreasing barriers such as displacement, the weather, or going to activities where there are many people, can generate some insecurity in some people and this option solves this problem [57].

The study has some limitations. Although the number of games and gamification processes for diabetes is growing, the number of studies analyzing its effect on HbA1c is low. Most of the studies come from the USA and all of them are from the Western countries, so the results should be taken into account with caution in countries with different culture and life style. Future research should analyze which factors promote the attractiveness of a game to create more enjoyable and desirable games. Additionally, in clinical practice, the implementation of the games with better results would be of interest for long term follow-up studies. Finally, the use of behavioral sciences [58] to identify the key information for the design and development of games for diabetes would be of interest for developing better games.

## 5. Conclusions

Games and gamification processes are beneficial for education in people with diabetes and to promote adherence to healthy lifestyle habits. However, their impact on glycated hemoglobin (HbA1c) does not appear to be clinically relevant. It is necessary to investigate into this area to find the mechanism to create more effective games for diabetes management and glycemic control.

## Figures and Tables

**Table 1:** Search equation information.

Search Equation Number	Search Equation Complete String
1	Diabetes AND (blood glucose OR HbA1c OR Glycated Hemoglobin A) AND (game OR games OR gamification OR virtual reality OR virtual environment OR video game OR video-game OR mobile game OR computer game OR serious game OR serious-games OR serious video- games OR serious games OR serious digital games OR serious electronic games OR serious gaming)
2	Diabetes AND (game OR games OR gamification OR virtual reality OR virtual environment OR video game OR video-game OR mobile game OR computer game OR serious game OR serious-games OR serious video-games OR serious games OR serious digital games OR serious electronic games OR serious gaming)

**Table 2:** Characteristics of the included studies (*n* = 10).

Author, Country (Year of Publication)	Study Design	Sample	Intervention	Main Results	EL/GR
Brown et al., USA (1997) [28].	Randomized controlled trial	*n* = 59 children (between 8 and 16 years old) with DM1 (28 in the control group and 31 in the intervention group).	Packy and Marlon game for the Super Nintendo. People must face diabetes risk by choosing healthy habits to stay healthy.	After 6 months: - Maintained HbA1c level. - Increased knowledge, communications with their parents and self care. - Decrease emergency visits. - Maintained HbA1c level.	1a/A
Cafazzo et al., Canada (2012) [23].	Pilot quasi-experimental study.	*n* = 20 adolescents (mean age 14.9) with DM1.	A mobile app receives glucometer readings. Points were gained for adhering to best-practice guidelines for blood glucose testing. Points and leveling up was rewarded with Apple Itunes and App Store purchases.	After 12 weeks: - HbA1c did not vary statistically. - Number of glucose reading increased. - High satisfaction with the intervention.	3b/B
Crawford & Wiltz, USA (2015) [27].	Case and control stduy	*n* = 411 people with diabetes (mean age 59.0)	Case group (*n* = 202 people with DM2). They play “Journey to Life Conversation Map”. Control group (*n* = 209). Usual care.	Case group HbA1c level decreased from 8.25% to 6.96%. Control group HbA1c level decreased from 8.57 to 8.27. *p* value between groups differences < than 0.001	3b/B
Dugas et al., USA (2018) [24].	Randomized clinical trial	*n* = 29 people with DM2 (mean age 65.4). (5 in the control group and 24 in the intervention group).	Smartphone app to improve glucose control, exercise, nutrition and medication adherence. Points were given to the patients if the followed healthy habits related with glucose control.	After 13 weeks: - HbA1c decreased from 8.66 to 8.06 in the intervention group and from 9.12 to 8.78 in the control group. - Improvement in treatment, diet and exercise adherence.	1a/A
Höschman et al., Switzerland (2019) [31].	Randomized controlled trial	*n* = 36 people with DM2 (mean age 57). (18 in the control group and 18 in the intervention group).	Smartphone game following physical activity guidelines to increase physical activity behavior. The use of the game and reaching the activity goals make the person progress in the storyline of the game.	After 24 weeks: - No statistically significant differences with the control group in HbA1c levels. - Increased steps number in the intervention group.	1a/A
Joshi et al., USA (2017) [30].	Randomized controlled trial	*n* = 97 people with DM2. (31 in the control group and 66 in the intervention group).	Game for Ipad with a 12-min duration. The game simulates multiple real-world pressures from job and family for people with diabetes including excuses for non-adherent health habits. The patients have to deal with those situations.	After 3 months: - HbA1c in the intervention group significantly decreased from 10.7 to 9.6 but no comparison was done with the control group. - The adherence to medication, diet and exercise significantly increased but no comparison was done with the control group.	1a/A
Joubert et al., France (2016) [29]	Cohorts study	*n* = 38 children with DM1 (mean age 13.7)	They used a game called “L’Affaire Birman” as an educative tool for insulin therapy. Children must solve the problems of the character (Alex) with his DM1	After 3 months: - HbA1c final level was not statistically different between the players and non-players. - Increased knowledge about insulin therapy and quantifying carbohydrates.	2b/B
Kempf and Martin, Deutschland (2013) [32].	Randomized controlled trial	*n* = 220 people with DM2 (mean age 61). (100 in the control group and 120 in the intervention group).	The intervention group received a Wii console with the game Wii Fit Plus and the balance board. They should use it 30 min a day for 12 weeks. Control group received usual care.	After 12 weeks: - HbA1c levels significantly decreased in the intervention group (6.8) but no significant differences were found with comparing with the control group (6.7). - Physical activity, fasting blood glucose, quality of life, depression and body mass index improved in the intervention group	1a/A
Kerfoot et al., USA (2017) [25].	Randomized controlled trial	*n* = 456 people with DM2 (mean age 59). (229 in the control group and 227 in the intervention group).	On-line game about diabetes self-management sends questions via e-mail or app to the patients about diabetes self-management. Points were earned if they answered correctly. The game made groups and individual and team scores were shown on a leaderboard.	After 12 months: - Differences in HbA1c were not significant between groups. - Differences in empowerment were not significant between groups.	
Kumar et al., USA (2004) [26].	Randomized controlled trial	*n* = 40 children (mean age 13.6) (21 in the control group and 19 in the intervention group).	Motivational game through PDA with diabetes management software. Patients were challenged to predict their glucose level and they earned points for being accurate and for playing the game.	After 4 months: - HbA1c levels did not differ between game and control group. - Hyperglycemia was less frequent in game group. - Game group knowledge improvement differed from control group	1a/A

Note: DM1 = Diabetes Mellitus type 1; DM2 = Diabetes Mellitus type 2; EL = evidence level; GR = grade of recommendation; HbA1c = glycated hemoglobin; PDA = Personal digital assistant.

## References

[1] Khan R.M.M., Chua Z.J.Y., Tan J.C., Yang Y., Liao Z., Zhao Y. (2019). From Pre-Diabetes to Diabetes: Diagnosis, Treatments and Translational Research. Medicina..

[2] Tong Y., Yang L., Shao F., Yan X., Li X., Huang G., Xiao Y., Zhou Z. (2020). Distinct secretion pattern of serum proinsulin in different types of diabetes. Ann. Transl. Med..

[3] American Diabetes Association (2018). Classification and Diagnosis of Diabetes: Standards of Medical Care in Diabetes. Diabetes Care.

[4] Chaterjee S., Khunti K., Davies M.J. (2017). Type 2 diabetes. Lancet.

[5] Farabi S.S., Hernandez T.L. (2019). Low-Carbohydrate Diets for Gestational Diabetes. Nutrients.

[6] World Health Organization (2016). Global Report on Diabetes. https://www.who.int/diabetes/global-report/es/.

[7] Ogurtsova K., Da Rocha J.D., Huang Y., Linnenkamp U., Guariguata L., Cho N.H., Cavan D., Shaw L.E., Makaroff L.E. (2017). IDF Diabetes Atlas: Global estimates for the prevalence of diabetes for 2015 and 2040. Diabetes Res. Clin. Pract..

[8] Crandall J.P., Polsky S., Howard A.A., Perreault L., Bray G.A., Barrett-Connor E., Brown-Friday J., Whittington T., Foo S., Ma Y. (2009). Alcohol consumption and diabetes risk in the diabetes prevention program. Am. J. Clin. Nutr..

[9] Brown S.A., García A.A., Brown A., Becker B.J., Conn V.S., Ramírez G., Winter M.A., Sumplin L.L., García T.J., Cuevas H.E. (2016). Biobehavioral determinants of glycemic control in type 2 diabetes: A systematic review and meta-analysis. Patient Educ. Couns..

[10] American Diabetes Association (2014). Standards of Medical Care in Diabetes. Diabetes Care.

[11] Moher D., Shamseer L., Clarke M., Ghersi D., Liberati A., Petticrew M., Shekelle P., Stewart L.A. (2015). Preferred reporting items for systematic review and meta-analysis protocols (PRISMA-P) 2015 statement. Syst. Rev..

[12] Roberts S., Barry E., Craig D., Airoldi M., Bevan G., Greenhalgh T. (2017). Preventing type 2 diabetes: Systematic review of studies of cost-effectiveness of lifestyle programmes and metformin, with and without screening, for pre-diabetes. BMJ Open.

[13] Dudlay B., Heiland B., Kohler-Raush E., Kovic M. (2014). Education and technology used to improve the quality of life for people with diabetes mellitus type II. J. Multidiscip. Health.

[14] Mohamed N.A.A., Huassain A.A.O. (2018). Impact of a multidisciplinary intensive education program on type 2 diabetes mellitus patients’ glycemic control and cardiovascular risk factors. Saudi Med. J..

[15] Thompson D., Baranowski T., Buday R. (2010). Conceptual Model for the Design of a Serious Video Game Promoting Self-Management among Youth with Type 1 Diabetes. J. Diabetes Sci. Technol..

[16] Padman R., Jaladi S., Kim S., Kumar S., Orbeta P., Rudolf K., Tran T. (2013). An evaluation framework and a pilot Study of a mobile platform for diabetes self-management: Insights from pediatric users. Stud. Health Technol. Inf..

[17] Sardi L., Idri A., Fernández-Alemán J.L. (2017). A systematic review of gamification in e-health. J. Biomed. Inf..

[18] Lazem S., Webster M., Holmes W., Wolf M. (2016). Games and Diabetes: A Review Investigating Theoretical Frameworks, Evaluation Methodologies, and Opportunities for Design Grounded in Learning Theories. J. Diabetes Sci. Technol..

[19] Dé Cássia V., Fels S., Castanheira L. (2017). The Value of Children’s Voices for a Video Game Development in the Context of Type 1 Diabetes: Focus Group Study. JMIR Diabetes.

[20] Moher D., Liberati A., Tetzlaff J., Altman D.G. (2009). Preferred reporting items for systematic reviews and meta-analyses: The PRISMA statement. PLoS Med..

[21] OCEBM Levels of Evidence Working Group The Oxford 2011 Levels of Evidence. Oxford Centre for Evidence-Based Medicine. http://www.cebm.net/wp-content/uploads/2014/06/CEBM-Levels-of-Evidence-2.1.pdf.

[22] Higgins J., Thomas J., Chandler J., Cumpston M., Li T., Page M., Welch V. (2019). Cochrane Handbook for Systematic Reviews of Interventions.

[23] Caffazo J.A., Casselman M., Hamming N., Katzman D.K., Palmer M.R. (2012). Design of an mHealth App for the Self-management of Adolescent Type 1 Diabetes: A Pilot Study. J. Med. Internet Res..

[24] Dugas M., Crowley K., Gao G.G., Xu T., Agarwal R., Kruglanski A.W., Steinle N. (2018). Individual differences in regulatory mode moderate the effectiveness of a pilot mHealth trial for diabetes management among older veterans. PLoS ONE.

[25] Kerfoot B.P., Gagnon D.R., McMahon G.T., Orlander J.D., Kurgansky K.E., Conlin P.R. (2017). A Team-Based Online GameImproves Blood Glucose Control in Veterans with Type 2 Diabetes: A Randomized Controlled Trial. Diabetes Care.

[26] Kumar V.S., Wentzell K.J., Mikkelsen T., Pentland A., Laffel L.M. (2004). The DAILY (Daily Automated Intensive Log for Youth) Trial: A Wireless, Portable System to Improve Adherence and Glycemic Control in Youth with Diabetes. Diabetes Technol. Ther..

[27] Crawford P., Wiltz S. (2015). Participation in the Journey to Life Conversation Map Improves Control of Hypertension, Diabetes, and Hypercholesterolemia. J. Am. Board Fam. Med..

[28] Brown S.J., Lieberman D.A., Gemeny B.A., Fan Y.C., Wilson D.M., Pasta D.J. (1997). Educational video game for juvenile diabetes: Results of a controlled trial. Med. Inf..

[29] Joubert M., Armand C., Morera J., Tokayeva L., Guillaume A., Reznik Y. (2016). Impact of a Serious Videogame Designed for Flexible Insulin Therapy on the Knowledge and Behaviors of Children with Type 1 Diabetes: The LUDIDIAB Pilot Study. Diabetes Technol. Ther..

[30] Joshi R., Joshi D., Cheriyath P. (2017). Improving adherence and outcomes in diabetic patients. Patient Prefer. Adherence.

[31] Hochsmann C., Muller O., Ambuhl M., Klenk C., Konigstein K., Infanger D., Walz S.P., Schmidt A. (2019). Novel Smartphone Game Improves Physical Activity Behavior in Type 2 Diabetes. Am. J. Prev. Med..

[32] Kempf K., Martin S. (2013). Autonomous exercise game use improves metabolic control and quality of life in type 2 diabetes patients—A randomized controlled trial. BMC Endocr. Disord..

[33] Charlier N., Zupancic N., Fieuws S., Denhaerynck K., Zaman B., Moons P. (2016). Serious games for improving knowledge and self-management in young people with chronic conditions: A systematic review and meta-analysis. J. Am. Med. Inform. Assoc..

[34] Ebrahimpour F., Najafi M., Sadeghi N. (2014). The Design and Development of a Computer Game on Insulin Injection. Electron. Physician.

[35] Marchetti D., Fraticelli F., Polcini F., Lato R., Pintaudi B., Nicolucci A., Fulcheri M., Mohn A., Chiarelli F., Di Vieste G. (2015). Preventing Adolescents’ Diabesity: Design, Development, and First Evaluation of “Gustavo in Gnam’s Planet”. Games Health J..

[36] Koivisto J., Malik A. (2020). Gamification for Older Adults: A Systematic Literature Review. Gerontologist.

[37] Johnson C., Feinglos M., Pereira K., Hassel N., Blascovich J., Nicollerat J., Beresford H.F., Levy J., Vorderstrasse A. (2014). Feasibility and Preliminary Effects of a Virtual Environment for Adults with Type 2 Diabetes: Pilot Study. JMIR Res. Protoc..

[38] Al Marshedi A., Wills G., Ranchhod A. (2016). Gamifying Self-Management of Chronic Illnesses: A Mixed-Methods Study. JMIR Serious Games.

[39] Markowitz J.T., Harrington K.R., Laffel L.M. (2013). Technology to Optimize Pediatric Diabetes Management and Outcomes. Curr. Diabetes Rep..

[40] Ruggiero L. (2015). Diabetes Prevention and Management: What does a Serious Game Have to Do with It?. Games Health J..

[41] Theng Y.L., Lee J.W.Y., Patindan P.V., Foo S.S.B. (2015). The Use of Videogames, Gamification, and Virtual Environments in the Self-Management of Diabetes: A Systematic Review of Evidence. Games Health J..

[42] Arthur L., Martins R., Alejandro P., Zonato R., Cristina I. (2015). User Assessment of “InsuOnLine” a Game to Fight Clinical Inertia in Diabetes: A Pilot Study. Games Health J..

[43] Swartwout E., El-Zein A., Deyo P., Sweenie R., Streisand R. (2016). Use of Gaming in Self-Management of Diabetes in Teens. Curr. Diabetes Rep..

[44] Larkin A.T., Hoffman C., Stevens A., Douglas A., Bloomgarden Z. (2015). Determinants of adherence to diabetes treatment. J. Diabetes.

[45] Martos M.B., Velando A., Pradas L., Suleiman N., Cañadas G.A., Albendín L., Gómez J.L. (2020). Smartphones and Apps to Control Glycosylated Hemoglobin (HbA1c) Level in Diabetes: A Systematic Review and Meta-Analysis. J. Clin. Med..

[46] Basiri R., Spicer M.T., Levenson C.W., Ormsbee M.J., Ledermann T., Arjmandi B.H. (2020). Nutritional Supplementation Concurrent with Nutrition Education Accelerates the Wound Healing Process in Patients with Diabetic Foot Ulcers. Biomedicines.

[47] Ramadas A., Chan C.K.Y., Oldenburg B., Hussein Z., Quek K.F. (2018). Randomised-controlled trial of a web-based dietary intervention for patients with type 2 diabetes: Changes in health cognitions and glycemic control. BMC Public Health.

[48] Ersotelos N., Margioris A.N., Zang X., Dong F. (2018). Review of mobile applications for optimizing the follow-up care of patients with diabetes. Hormones.

[49] Valenzuela T., Okubo Y., Woodbury A., Lord S.R., Delbaere K. (2018). Adherence to Technology-Based Exercise Programs in Older Adults: A Systematic Review. J. Geriatr. Phys. Ther..

[50] Halbrook Y.J., O’Donnell A.T., Msetfi R.M. (2019). When and how video games can be good: A review of the positive effects of video games on well-being. Perspect. Psychol. Sci..

[51] Arnold J.C. (2020). The Importance of Different Knowledge Types in Health-Related Decisions—The Example of Type 2 Diabetes. Sustainability.

[52] Maillot P., Perrot A., Hartley A., Do M.C. (2014). The braking force in walking: Age-related differences and improvement in older adults with exergame training. J. Aging Phys. Act..

[53] Li J., Theng Y.L., Foo S. (2016). Effect of exergames on depression: A systematic review and meta-analysis. Cyberpsychol. Behav. Soc. Netw..

[54] Brawley L.R., Rejeski W.J., King A.C. (2003). Promoting physical activity for older adults. The challenges for changing behavior. Am. J. Prev. Med..

[55] Keogh J.W.L., Power N., Wooller L., Lucas P., Whatman C. (2014). Physical and psychosocial function in residential aged-care elders: Effect of Nintendo Wii Sports games. J. Aging Phys. Act..

[56] Douris C.P., McDonald C.B., Vespi C.F., Kelley C.N., Herman C.L. (2012). Comparison between Nintendo Wii Fit aerobics and traditional aerobic exercise in sedentary young adults. J. Strength Cond. Res..

[57] Yardley L., Bishop F.L., Beyer N., Hauer K., Kempen G., Piot-Ziegler C., Todd C.J., Cuttelod T., Horne M., Lanta K. (2006). Older people’s views of falls-prevention interventions in six European countries. Gerontologist.

[58] Thompson D., Baranowski T., Buday R., Baranowski J., Thompson V., Jago R., Griffit M.J. (2010). Serious video games for health how behavioral science guided the development of a serious video game. Simul. Gaming.

